# Cancer patients’ experiences and preferences when receiving bad news: a qualitative study

**DOI:** 10.1007/s00432-022-04311-8

**Published:** 2022-08-23

**Authors:** Theresia Krieger, Sandra Salm, Antje Dresen, Natalia Cecon

**Affiliations:** Faculty of Medicine and University Hospital Cologne, Faculty of Human Sciences, Institute for Medical Sociology, Health Services Research, and Rehabilitation Science (IMVR), University of Cologne, Eupener Str. 129, 50933 Cologne, Germany

**Keywords:** Cancer, Receiving bad news, Patient experiences, Physician–patient communication, Patient preferences, Qualitative research

## Abstract

**Purpose:**

Receiving a cancer diagnosis significantly impacts patients’ lives, and how the bad news is delivered influences patients’ trajectory, psychosocial adjustment and openness to psycho-oncological support. We explored how patients’ experiences, reactions and preferences were when receiving the news and which optimization recommendations can be made.

**Methods:**

We conducted an exploratory qualitative study with patients who enrolled in the new integrated cross-sectoral psycho-oncological care programme ‘isPO’, being enrolled 12 months post-diagnosis. Data on the main issue (i.e. perception of the moment when the diagnosis is received) were collected via telephone interviews that were fully audiotaped and transcribed. Two independent coders conducted inductive content analyses using MAXQDA.

**Results:**

Out of 38 approached patients, 23 cancer patients with 13 different tumour entities participated. They had a mean age of 54.2 (SD 16.2); *n* = 17 (74%) were female. Three major themes with 14 corresponding subthemes emerged: (1) patients’ experiences with the bad news delivery, including setting, mode, preparation and perceived needs; (2) patients’ reactions to the bad news, such as shock, fear and helplessness, disbelief and denial, anger and feeling of injustice, thankfulness and depression; and (3) patients’ receiving preferences, including psycho-oncological support, addressing informational needs, needs-driven comprehensive support and a competent multidisciplinary support team.

**Conclusions:**

The quality of bad news delivery and addressing patients’ needs should be strongly considered by physicians. We recommend integrating patients’ perspective on the quality management processes of breaking bad news. For providing needs-centred high-quality care, applying existing guidelines and acquiring patient-centred communication skills are central.

## Introduction

### Delivering bad news

Receiving a cancer diagnosis is perceived as life-threatening by many patients, regardless of at which stage it is diagnosed (Hagerty et al. 2005). Cancer is associated with several potentially negative events, such as pain, loss of physical function, negative treatment effects and death (Mazzocco et al. 2019; Meneguin et al. 2018; Anuk et al. 2022).

In the clinical routine, breaking bad news (BBN) is a frequent duty that remains a communicative challenge (Schilling xxxx). This moment changes a patient’s view of the future significantly (Buckman 1984). Physicians’ communication skills, attitudes and delivery modes impact patients’ decision-making and adherence to treatment (Zachariae et al. 2003; Sobczak et al. 2018). Delivering and receiving bad news is considered stressful for both physicians and patients (Ptacek and Eberhardt 1996). Physicians may develop physiological stress responses and anxiety, particularly when they assess the conversation as ‘unsatisfactory’ (Shaw et al. 2013; Friedrichsen and Milberg 2006). Especially for patients, as recipients of bad news, this moment is often highly emotional and overwhelming (Matthews et al. 2020; Monden et al. 2016). Retrospectively, patients and their families described the BBN situation as a ‘turning point’ or biographical caesura (Bumb et al. 2017). In a German study, not even half of the cancer patients were ‘completely satisfied’ with their BBN experience (Seifart et al. 2014). From patients’ perspective, deficiencies are perceived by the amount of time given for the news delivery, physicians’ attention to the topic, comprehensibility of the news (e.g. frequent use of medical terminology) as well as emotional and cognitive support needs. (Sobczak et al. 2018)

BBN demands effective patient-centred communications skills, professionalism, patient engagement strategies, empathy and patience (Sobczak et al. 2018; Rosenzweig 2012; Baile and Aaron 2005; Thistlethwaite 2009). Ineffective delivery may strongly impact patients’ stress and anxiety, adjustment to the diagnosis, coping and openness to psycho-oncological support (Ptacek and Eberhardt 1996; Fallowfield and Jenkins 1999). Therefore, specific communication skills, competences and experiences are considered crucial for adequate BBN. (Buckman 1984; Rosenzweig 2012; Thistlethwaite 2009)

In a survey of physicians, the majority consider BBN skills to be very important, but only 40% felt they had the necessary training to deliver such news successfully (Monden et al. 2016). Communication training significantly improves attending physicians’ attention to patient responses after BBN, enhances their capacity to address feelings and communication-related emotions, and augments their active listening skills—all of which lead to significant improvements in patient adherence to treatment (Gorniewicz et al. 2017; Zolnierek and DiMatteo 2009). Most physicians state that BBN situations require comprehensive, formal training for skill development (Alelwani and Ahmed 2014; Karger et al. 2017). A number of evidence-based recommendations and guidelines for BBN (e.g. SPIKES or ABCDE (Baile et al. 2000; Rabow and McPhee 1999)) offer training and further education formats that may assist physicians. Before the news delivery, patients’ communication preferences can be assessed by applying the Marburg Breaking Bad News Scale (MABBAN), which is a SPIKES-protocol questionnaire (Blanckenburg et al. 2020). Using a communication protocol can also increase or safeguard the quality of communication during the BBN (Sobczak et al. 2018).

Acquiring BBN skills early in one’s medical career is important, and video-based work examples have been shown to be helpful (Schmitz et al. 2020). However, not all German medical school curricula include specific skills training for BBN in cancer (Berney et al. 2017). In several oncological settings, BBN capacity building for physicians is offered, promoted or piloted on a voluntary basis (Vitinius et al. 2013; Ernstmann et al. 2022), mostly applying the SPIKES protocol (Baile et al. 2000). Furthermore, research on how many physicians participate in the training and apply these skills is scarce but would be necessary in order to assess BBN quality. For a comprehensive understanding of the status-quo, it would be helpful to explore physicians’ adherence to these guidelines from the patients’ perspective (Sobczak et al. 2018; Seifart et al. 2014).

### Objective

Although various international studies have investigated patients’ experiences and their preferences regarding receiving bad news (Meneguin et al. 2018; Ptacek and Eberhardt 1996; Matthews et al. 2020), this topic remains insufficiently explored in Germany (Seifart et al. 2014). Moreover, by gaining a comprehensive understanding of patients’ needs and acknowledging their experiences, physicians may be better equipped to break bad news in a supportive way.

The primary aim of this study is to deeply explore cancer patients’ subjective experiences, reactions and preferences when receiving the bad news of their cancer diagnosis. Based on this comprehensive understanding we make recommendations for optimizing the BBN process.

## Methods

### Setting

In Germany, the integrated, cross-sectoral psycho-oncological care programme *‘isPO’* was designed, implemented and evaluated between 2017 and 2022 (Jenniches et al. 2020). The programme is offered to newly diagnosed adult cancer patients for 12 months, parallel to their biomedical treatment (Kusch et al. 2022). On an individual level, isPO aims to reduce patients’ symptom severity of their depression and anxiety; on a system level, it offers a needs-driven psycho-oncological care programme for comprehensive implementation into nationwide cancer care (Jenniches et al. 2020). The programme was implemented in January 2019 in four specially established psycho-oncological care networks in North Rhine-Westphalia, Germany.

Furthermore, isPO is externally evaluated by an independent institute that continuously applies a mixed-methods approach (Jenniches et al. 2020). Part of the external summative evaluation was to assess the programme’s quality of care, for which purpose qualitative and quantitative data from patients and isPO service providers were collected (Krieger et al. 2021). Within the evaluation of the isPO programme, research was conducted by four researchers: a public health expert/nurse, a health services researcher/speech & language therapist, a sociologist/speech teacher, and a psychologist. All four researchers were female. For the presented research objective on patients’ experiences with BBN, we used qualitative data collected during the summative evaluation. All patients included in this study received psycho-oncological support through isPO. In the course of exploring both the effect and quality of care of the isPO programme (Jenniches et al. 2020), we identified the moment of BBN to a patient as a ‘starting point’ for all further biomedical treatment and psycho-oncological care (Krieger et al. 2021).

### Study design and ethics

This qualitative explorative interview study was conducted with cancer patients who enrolled in the isPO programme between April 2020 and March 2021. Relevant national and European data protection regulations were obeyed during data collection, and patients’ anonymity and confidentiality were protected at all times. Participants received no compensation.

### Sampling and enrolment procedure

To identify and select information-rich cases for the most effective use of limited resources, purposeful sampling—a nonprobability sampling technique—was applied (Denscombe 2005). Purposeful sampling is defined as ‘intentional selection of informants based on their ability to elucidate a specific theme, concept, or phenomenon’ (Robinson and Michalos 2014). In our case, only isPO patients were included in this study. To gain an in-depth understanding, further inclusion criteria were: (1) considering patients from all four isPO study sites as well as different (2) sexes, (3) ages and (4) tumour entities. Preconditions for their participation were cancer patients’ accessibility, availability and willingness to participate in an interview. Exclusion criteria included factors associated with patients’ state that might have made it difficult to set up or conduct an interview (e.g. cognitive impairments, pain, speech problems).

Patients were enrolled after finalizing their individual isPO trajectory (> 12 months post-diagnosis). The enrolment process was initiated by professionals from the isPO care team (e.g. isPO case manager or psychotherapist) during the final counselling session by informing patients about a possible interview participation. If patients expressed interest, they were contacted by researchers from the especially established isPO Trust Centre. If positive oral consent was given, a date for the interview was arranged and a written consent form was sent to the prospective participant. Due to the ongoing COVID-19 pandemic, face-to-face interviews with patients were avoided. Instead, interviews were conducted via telephone after receiving the signed consent forms.

### Data collection

The interview guideline was developed by the interdisciplinary team. Before its application, it was piloted with three cancer survivors from the project stakeholder, House of the Cancer Patient Support Associations of Germany (HKSH-BV). Semi-structured telephone interviews were conducted by the research team. Notes were taken during data collection. The main goal of these interviews was to explore how patients experienced the isPO programme, starting with the question about the BBN (in terms of their cancer diagnosis). However, for this study’s explorative purpose, only the opening parts of the interview were utilized. The initial narrative question was: ‘Could you describe how you perceived the moment of receiving the diagnosis?’, followed by deepening questions (e.g. ‘How was the mode or mode of transmission?’, ‘How did you feel?’). Thus, the patient’s individual experiences, reactions, and needs or preferences were explored.

### Data analysis

Audiotaped data were fully transcribed by an external transcription bureau whilst considering the standards for social research (Dresing et al. 2018). Transcripts were anonymized by the research team. First, each transcript was labelled with an interview ID number. Next, all possible identifiers were removed from the transcripts (e.g. names of professionals, study sites or hospitals). Two researchers coded the transcripts independently by applying inductive content analysis (Graneheim and Lundman 2004; Mayring xxxx) and by using MAXQDA 2018. Material and inductive codes were discussed between the two researchers with the aim of achieving a profound understanding of the patients’ individual experiences and preferences. The entire process was critically accompanied by discussions among the coders, which continued until a consensus about how to group the findings was reached, whereupon a final coding system was agreed to. Themes and subthemes were condensed, and representative quotes were extracted. Data collection and analyses continued until a rich description of patients’ experiences was obtained.

## Results

### Sample characteristics

At the end of their isPO care trajectory, 38 patients were approached; 23 agreed to participate, and 15 declined. Reasons for not participating in an interview included ongoing cancer treatment, suffering from a physical ailment (e.g. fatigue) or feelings of insufficient emotional stability to participate in an interview.

Participants’ ages varied between 33 and 65 years (mean 54 years); *n* = 17 (74%) were female and the majority *n* = 17 (74%) were employed. Patients from all four isPO care networks shared their experiences. The sample included patients with 13 different tumour entities, among which breast cancer was the most prominent (30%). Table 1 depicts patient characteristics in detail.

**Table 1:** Patient characteristics

Characteristics	*N*	%	Mean (SD)
Age			54.2 (16.2)
Sex	23	100	
Male	6	26	
Female	17	74	
Occupational status	23	100	
Employed	17	74	
Part-time employed	1	4.3	
Retired	3	13	
Early retired	1	4.3	
Studying	1	4.3	
Not employed	1	4.3	
isPO care network	23	100	
Network 1	9	39.1	
Network 2	4	17.4	
Network 3	5	21.7	
Network 4	5	21.7	
Tumour entity	23	100	
Breast	7	30.4	
Rectum & Colon	4	17.2	
Prostate	2	8.6	
Thyroid gland	2	8.6	
Other (Kidney, Melanoma, Non-Hodgkin lymphoma, Skin, Uterus, Bronchia, Parotid gland, Unspecified)	8	34.8	

The total interview material comprises 21 h and 40 min; the part of the interview concerning patients’ BBN experiences was approximately 20 min per interview (in total, approx. 7 h, 40 min).

### Patients’ experiences and preferences

**Fig. 1:**
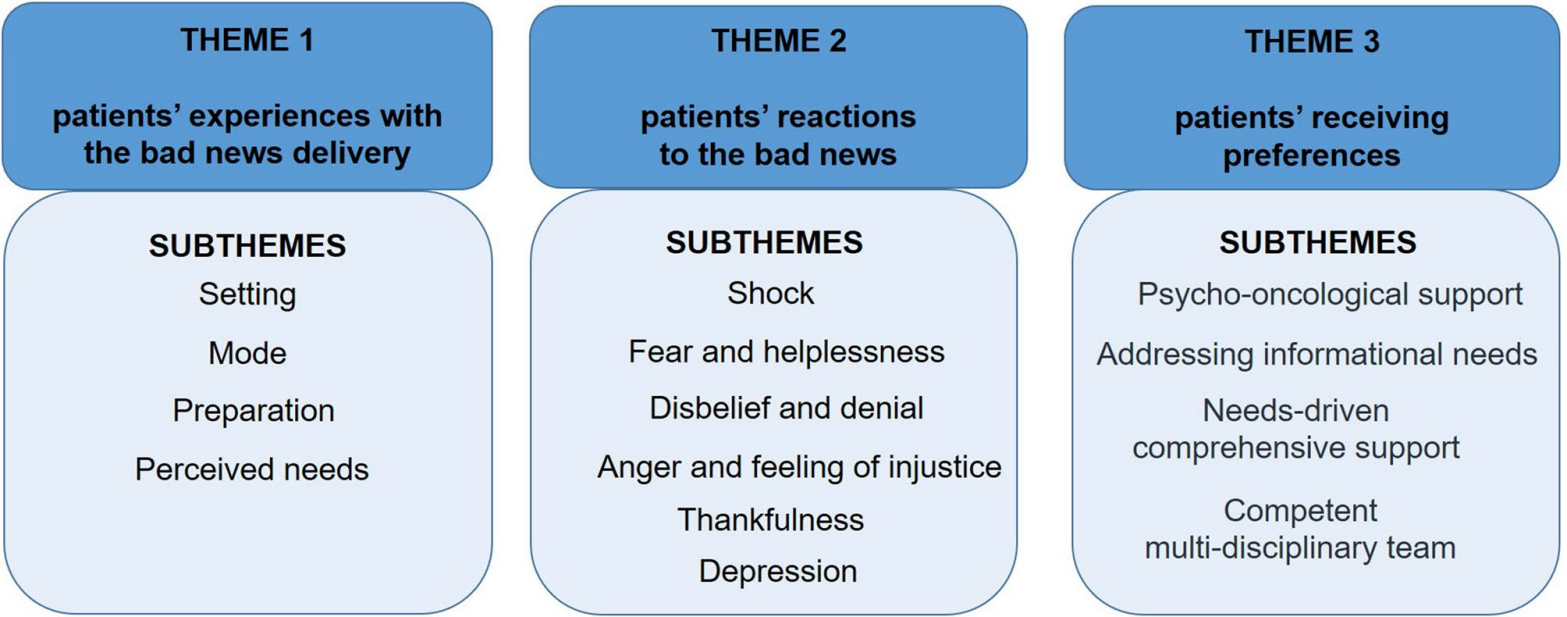
Emerging themes and subthemes when receiving bad news

According to the research objective, 231 quotes were identified from the material. Three major themes emerged: (1) patients’ experiences with the bad news delivery (76 quotes), patients’ reactions to bad news (131 quotes) and (3) patients’ receiving preferences (24 quotes). Figure 1 illustrates these themes along with their corresponding subthemes; Tables 2, 3 and 4 illustrate these subthemes using example quotes.

**Table 2:** isPO patients’ experiences of bad news delivery with subthemes

Subthemes with findings	Examples
Setting	
Receiving the bad news alone	‘So that day, this doctor didn't even ask whether I was there alone or if I could call someone to pick me up. In hindsight, I'm glad that I didn't drive there, but took the train instead. I don't know how I got home. So I just don't know anymore, right? And then I had somehow just black around me’. [ID6]
Receiving the bad news with a partner	‘My husband was with me. Sure, the emotions then attacked both of us. The doctor also said that we should talk together’. [ID16]
Mode	
Face-to-face	‘I was sitting with the doctor in his room and he was talking to me very clearly. I could see in his eyes that it was serious’. [ID5]
Via telephone	‘I got the diagnosis in a very, very short phone call and then had this moment when I hung up and first thought: “No, that can't be”. Suddenly, someone comes along who really takes just 30 s to say something like that, and I felt very lost at that moment’. [ID11]
Preparation	
Unexpected	‘It came totally unexpected. Yes, it's just great that you can do all your check-ups and that the doctors—or, at least, my gynaecologist—was very attentive and said “Here, you have to be there. I don’t know, that's it. Let everything be checked”. So, then it came out’. [ID15]
Forewarned	‘I first went to the gynaecologist. It was just for a routine check-up. I had a breast ultrasound done and then the lump was discovered. The gynaecologist wasn’t sure whether it was benign or malign. But to be honest, I expected it to end badly because my mother died of breast cancer at the age of 38. That's why I had a mammogram and the biopsy in the breast centre. After three days, I got the diagnosis, and I was expecting it’. [ID21]
Perceived needs	
Communication style	‘So of course, it was a shocking situation. But [doctor’s name], my urologist, explained it to me very precisely and also without making a fuss, so neither/ How should I put it? Neither with pity nor as a disaster scenario’. [ID17]
Adequate information provision	‘I had a lot of questions that I couldn't ask anyone at that moment. That was just a feeling of not being able to believe it and of being overwhelmed’. [ID11]
Second opinion	‘I always tried to get a kind of second opinion, so to speak, and the good thing was that I was able to check the diagnoses with this cancer research centre in [city name], which I contacted by e-mail, and I could then always check the diagnoses with them so that it was quite useful for me as a second opinion and in the sense of, I say, counter-checking the knowledge’. [ID1]
Stepped approach	‘After the first shock, my doctor then put me in a room where I drank some water. Later, with my wife in the room, I was a bit less tense’. [ID20]
Shared decision-making	‘The senior physician explained both options. Then he said that we would have to decide the best option for me. He said that he could help us make a sound decision’. [ID15]
Structured treatment offer	‘There was a crystal-clear structure. I was released from inpatient treatment in December. Then, in January, the rat tail* started with chemotherapy and radiotherapy. You immediately had the feeling that it was being dealt with’. [ID18]
*’to entail a rat tail’ is a German figure of speech that can be understood as a chain reaction of problems or negative consequences that began or were created

**Table 3:** isPO patients’ reactions to bad news with subthemes

Subthemes	Examples
Shock	‘It shook me to my core, so it was really a very shocking experience’. [ID14]
Fear and helplessness	‘I understood that it is a rapidly growing and very aggressive tumour. It made me very, very scared that I had a very advanced stage and of course that got me down. I thought I wasn’t going to be healed and that it was already too late!’[ID8]
Disbelief and denial	‘Especially on the same day, and the days afterward, I had the feeling that it was about a different person – not about me. So we kept talking about someone else who got this diagnosis’. [ID2]
Anger and feeling of injustice	‘I don’t know the cancer! I didn’t want it! I didn’t invite it!’ [ID16]
Thankfulness	‘Everyone said in the meantime, I was lucky in my misfortune because if I hadn’t gotten appendicitis, I probably wouldn’t even know today that I had such a bastard sitting on the appendix?’ [ID16]
Depression	‘I've always described it like this: The ground opened up and I was way, way down. I fell in there and didn't see any daylight at all. So, it was practically all black around me. It took me about four weeks from the diagnosis to the operation, and during those four weeks I actually only had one topic in my mind, so I was just circling around this one topic’. [ID6]

**Table 4:** isPO patients’ preferences for receiving bad news (with subthemes)

Subthemes	Examples
Psycho-oncological support	‘There must be some way that I can have a conversation with someone. I have so many questions – I am so scared. The physician then said, “Yes, we have a psycho-oncologist here who looks after our patients here on site”’. [ID19]
Addressing informational needs	‘I think if you're addressed directly as a patient by an oncologist and as a patient you have to know that this support is due and offered to you. I think you perceive that better and more personally than if you just get an email, flyer or something else’. [ID2]
Needs-driven comprehensive support	‘The whole package, whether it was conversations with the psycho-oncologist, with the specialist nurse and then the whole staff in the ward of [doctor’s name] and that was great for me, every time we had something or discussed a topic or privately, it was completely open, humane’. [ID16]
Competent multidisciplinary team	‘The conversation actually calmed me down in the sense that you don't have to guess what happened. They put all the facts on the table, and now we have to find a way to deal with them’. [ID16]

#### Theme 1: patients’ experiences with bad news delivery

Four subthemes emerged: setting, mode, preparation and perceived needs (Table 2).

### Setting

At the time of the data collection, most patients received their bad news alone, which may have been due to the COVID-19 pandemic. In some cases, this was experienced as very ‘harmful’ [ID6] as patients felt alone with the dreadful news and regretted that they could not share the BBN experience with someone in their family. Many patients articulated that it was the first time that they had felt as though they were ‘losing control’ of their life and that they felt very vulnerable—even paralysed. Patients who received their bad news in the presence of their partners reported such accompaniment as being very helpful.

### Mode

Two BBN modes were identified, namely: face-to-face and by telephone. Patients receiving the news by telephone were negatively affected by this approach, reporting that they were unable to adequately process the news, which provoked feelings of ‘helplessness’ [ID11].

### Preparation

The majority of the interviewees received their diagnosis completely *‘unprepared’* [ID15], such as in cases where the diagnosis arose from a routine examination or routine operation (e.g. appendectomy). However, some patients had already received a few signals or ‘warnings’ [ID16] in advance, which made them ‘prick up their ears’ [ID15]—for example, when routine examinations were followed by more in-depth examinations by specialists. Additionally, some patients were sensitive to the topic due to a familial disposition.

### Perceived needs

Communication style, adequate information provision, second opinion, stepped provision, shared decision-making and a structured treatment offer were identified as ‘needs’ by patients.

Patients highlighted the ‘manner’ [ID20] of how the message was communicated as vital: ‘Constructive information’ [ID18]—paired with calm, clear transmission as well as reassuring and ‘future-oriented words’—was perceived as most helpful.

Patients articulated many informational needs (e.g. about the trajectory of treatment, social or psychological support). They required ‘Understandable information’ [ID11] in oral and written form. Information should be valid and ‘easily accessible’ [ID8]. However, immediately after the BBN, many felt overwhelmed and unable to absorb information.

Moreover, patients appreciated having the chance to ‘ask for a second opinion’ [ID3]. This was especially notable when the diagnosis or possible treatment options (e.g. chemotherapy vs. radiotherapy) were not yet clear. This approach augmented patients’ ‘confidence’ [ID11] in the care system.

Some patients reported that they could handle the situation better when the news was ‘delivered in two steps’ [ID6] and thereby having sufficient time to adapt to it. Others were challenged by the ‘uncertainty’ [ID11] when waiting for the next appointment.

Some patients reported that several treatment paths were explained to them during the BBN and that, with this knowledge, they felt empowered to choose their own ‘path’ [ID2]. Most appreciated that this decision was not made for them but rather in a ‘shared-decision manner’ [ID15] with the physicians.

Patients said that once the treatment decision was made, they received a trajectory timetable or personalized plan. Most observed that doctors made professional, structured and rapid appointments. Patients experienced *‘Structured treatment offers’* [ID1] as being crucial in the process of preparing themselves.

#### Theme 2: patients’ reaction to bad news

Six subthemes were detected: shock, fear and helplessness, disbelief and denial, anger and feeling of injustice, thankfulness, and depression (Table 3).

### Shock

Every interviewed patient experienced their cancer diagnosis as a shock. They described that it ‘pulls the rug out from underneath you’ [ID22] and ‘like a smack on the head’ [ID15]. Some described dissociative states like ‘getting only snippets’ [ID23] of the BBN conversation or that the conversation ‘ran like a waterfall past me’ [ID1] and one patient describes it as ‘sitting there […] and not getting all the things from the outside anymore’ [ID22]. Patients declared that they felt overwhelmed, empty, defeated, overburdened, dissolved (‘being in the wrong film’ [ID20]), horrified (‘as if a tank had rolled over me’ [ID10]) or totally lost.

In the following days, several patients described that they remained in a ‘rigid state’ or felt as though they had ‘fallen into a deep hole’ [ID22] and were ‘living in a vacuum’ [ID3]. Some mentioned that they could not stop crying. Others described a mental breakdown. The uncertainty in the beginning, when specific diagnostics were still incomplete, was ‘hard to endure’ [ID18].

Retrospectively, most patients described the diagnosis as a deep biographic incision that inclined them to question everything. ‘I thought that my life was destroyed. You no longer function as a woman, you’re just sick. What are you doing here?’. [ID18] Patients with a family or small children described this feeling as especially prominent. Patients who had to stop working also experienced this biographic incision very strongly.

### Fear and helplessness

All patients experienced fear at the moment of their diagnosis. However, different fear types were observed. Some patients were afraid of ‘not winning the fight’ [ID8] against cancer. Others mentioned that they were scared of ‘not having the chance to see their child growing up [ID18]’ and that it was ‘most terrifying of all’ [ID6] to apprehend the stress that their diagnoses provoked within the family, especially their children.

Also, anxiety regarding the therapy itself, such as specific examinations, negative side effects (e.g. pain, disability) or ineffective treatments was experienced. Some patients were preoccupied with the fear of ‘losing the ability to work’ [ID8].

Many patients felt helpless and reported that ‘being a very vulnerable person from one moment to the other’ [ID8] was like ‘losing control’ [ID21] and ‘[losing] the guarantee of a future’ [ID18].

### Disbelief and denial

Some patients needed time to ‘believe’ [ID18], ‘realize’ [ID23] and ‘accept’ [ID21] the diagnosis. They clung to the hope that the ‘diagnosis was a mistake’ [ID2] or misunderstanding. Some tried to distract themselves.

### Anger and feeling of injustice

Some patients felt angry because they felt ‘helpless’ [ID10]. However, they distinguished different anger subjects, such as that ‘the cancer was diagnosed so late’ [ID16], their families’ attempts to talk to them about the diagnosis, the feeling of injustice because ‘no one had cancer in the family’ [ID16] or that they were ‘living very healthily and consciously’ [ID21]. It was experienced as ‘unfair’ [ID21] that, besides having cancer, one also had to endure severe side effects like ‘losing all hair’ [ID18] and not being able ‘to have children’ [ID18].

### Thankfulness

Only one person reported feeling thankful for ‘the life so far’ [ID23]. Others’ thankfulness derived from the point that ‘the diagnoses was made early enough to be able to treat’ [ID13] or ‘feeling lucky’ [ID9] about accidental diagnoses.

### Depression

Nearly all interviewed patients experienced depressive moments, but these differed in severity. ‘lack of drive’ [ID10] and experiencing the diagnosis as ‘a big, deep, black hole’ [ID19] were often reported.

Many patients experienced sadness and apathy. As one patient described, ‘There was a phase, when all was always at such a low level, no feelings of great joy. Everything just rippled along so trivially’ [ID23].

Some patients reacted with social withdrawal from their family and friends, spending the days ‘just sitting there and crying’ [ID10] or being ‘very impolite’ [ID10] or aggressive to others or to themselves. Others experienced ‘hopelessness’ [ID9] and even latent suicidal thoughts. Descriptions ranged from perceiving ‘life chance as fifty-fifty’ [ID14] and losing the will to live, ‘that it will not have a good end’ [ID4], ‘seeing no sense in fighting’ [ID14] or ‘seeing all black’ [ID22]. One even mentioned that they would have preferred to commit suicide ‘if they did not have a family’ [ID21].

#### Theme 3: patients’ preferences for receiving bad news

Patients articulated four preferences for BBN: psycho-oncological support, addressing informational needs, needs-driven comprehensive support and a competent support team (Table 4).

### Psycho-oncological support

Patients proposed that doctors should include psychological support much earlier or even invite psycho-oncologists to the BBN conversation. Support should be offered immediately and as long as individually needed. Patients preferred a flexible transmission mode (e.g. face-to-face, telephone or video call); the timeframe within which psycho-oncological support is needed may vary as well. They mentioned that to meet their needs, sufficient psycho-oncological staff ‘should be made available’ [ID2]. One stated that psycho-oncology ‘should be integrated into routine cancer care’ [ID9].

### Addressing informational needs

Patients experienced ‘a huge thirst for information’ [ID19] and preferred to have their information needs adequately addressed (patient-centred). They favoured a ‘direct and personal conversation’ [ID2] with a dedicated professional (e.g. doctor in charge of their treatment). Furthermore, there was a preference to ‘receive guidance’ [ID19] in going through the different inpatient information materials (e.g. web-based patient information portals or flyers). Connecting patients to further outpatient support or care offers (e.g. national cancer care network) was also desired.

### Needs-driven comprehensive support

Patients specified that needs-based support and treatment would be helpful. This may include adequate pain therapy, other complementary medical approaches (e.g. acupuncture) or greater flexibility in the interaction and cooperation of different disciplines to connect the necessary treatments instead of separating them (e.g. nutritional therapeutic support and chemotherapy). Therefore, patients preferred comprehensive interdisciplinary care: ‘We need the “whole package” actually, whether it is the talk with the psycho-oncologist or with the specialist nurse and then the whole medical staff’ [ID19]. Patients wished to be treated ‘completely openly’ [ID15] in’a human way’ [ID15] with a ‘holistic approach’ [ID18].

### Competent multidisciplinary support team

Patients emphasized that it is important to have a ‘competent multidisciplinary team’ [ID16] accessible when receiving bad news. Especially in the first days, when patients feel ‘a bit lost’ [ID1], offering careful and structured orientation (e.g. timeline, roles, and procedures) is desired. In addition, addressing the issues of difficulties during treatment or treatment errors were raised. ‘Open’ [ID15], evidence-based and ‘empathic’ [ID2] patient-centred communication was endorsed.

## Discussion

This study explored patients’ experiences with receiving their cancer diagnosis, their reactions to it, and their preferences for receiving the ‘bad news’ of their cancer diagnosis within a setting where the new isPO programme was offered. Findings illustrate the multifaceted task of BBN and reflect the emotionally complexity of patients’ reactions and what negative effects it can have on their psyche, their environment and their lives. (Matthews et al. 2020; Thistlethwaite 2009)

### Patients’ experiences receiving bad news

#### The delivery of bad news

In this exploratory study, although we did not explicitly focus on SPIKES (‘Setting up’, ‘Perception’, ‘Invitation’, ‘Knowledge’, ‘Emotions with empathy’ and ‘Strategy’ (Baile et al. 2000)), our patients did address some SPIKES components. Concerning ‘Setting up’, some patients articulated positive experiences such as having a face-to-face conversation. One of patients’ most negative BBN experiences was receiving bad news via telephone. This should be avoided whenever possible because it allows no visual feedback on how the patient reacts to the news and patients may not have time to ask questions (Thistlethwaite 2009). Furthermore, ‘preparing’ the patient—such as by arranging a safe atmosphere and allocating sufficient time—appeared to be important, (e.g. via a stepped communication approach) to facilitate the process when the bad news is broken ‘unexpectedly’ (Rabow and McPhee 1999). Within the perceived needs, the SPIKES component of ‘knowledge’ (e.g. adequate information provision) became especially evident (Alelwani and Ahmed 2014; Baile et al. 2000). To make informed decisions, our findings show that patients require ‘accurate’ and patient-centred communication (Rosenzweig 2012; Singh and Agarwal 2017). Finally, the component of ‘strategy’ was highlighted as a requirement—in particular, patients appreciated receiving a clear ‘treatment time table’ (Rosenzweig 2012; Baile et al. 2000).

#### Patients’ reaction to bad news

Our findings particularly illustrate that the BBN situation was a ‘decisive moment’ for patients and that its quality had an immediate and lasting effect on patients’ psychological well-being as well as on their utilization of services (e.g. psycho-oncological support), corroborating the findings of other studies (Hagerty et al. 2005; Sobczak et al. 2018; Matthews et al. 2020). It is recognized that cancer diagnoses cause psychological problems and that cancer patients have a higher suicide risk than the general population (Madeira et al. 2011). In our setting, it remained unclear whether—and how many—oncologists are qualified for these complications. Moreover, it appears to be important to consider that patients also experience trauma-associated symptoms (e.g. dissociation) and that some patients may not be able to emotionally express their need for support. To avoid a deterioration in patients’ mental well-being, physicians should be capable of identifying emotional reactions and exploring support needs for coping with the illness; or they should transfer the patient to professional psycho-oncological care as early as possible. This implies a holistic health care approach (Matthews et al. 2020; Beck et al. 2002) that incorporates psycho-oncological care offers into the standard care for cancer patients.

### Preferences for receiving bad news

The perceived manner, attitude and quality of the BBN conversation are recognized as critical factors in shaping patients’ decision-making ability and future orientation (Sobczak et al. 2018). Therefore, it is important to know patients’ needs and future expectations (Baile and Aaron 2005; Baile et al. 2000). All interviewed patients received professional psycho-oncological care as proposed by the national cancer guideline amongst others (Institute of Medicine 2008; Bundesminiterium für Gesundheit), and all confirmed their preference for receiving such care. The findings regarding preferences when receiving bad news are similar to the results of other studies (Sobczak et al. 2018; Beck et al. 2002). Our findings show that patients had various information needs that were not always met. To sensitize health care providers to patients’ needs, we recommend investment in specific training programmes, such as KoMPASS (Karger et al. 2017). In this respect, addressing on patients’ context specific experiences and building on their needs appears crucial. We are in line with Beck et al. (2002) in that we consider it essential to apply patient-friendly language and questioning techniques as well as to provide sufficient time for questions, information, summaries and clarification (Beck et al. 2002).

### Study limitation

Some limitations in our study should be noted. First, this study included only isPO patients who had received psycho-oncological support since the beginning of their trajectory. We applied this nonprobability sampling technique by enrolling only a’hand-picked’ sample because our research did not aim to generate results that would be generalisable to all cancer patients (Denscombe 2005). Therefore, any such generalisation of the findings or comparison to patients who did not enrol in isPO is limited. Second, only patients who ‘felt ready’ to share their perspectives were included, which may have resulted in a bias. Third, we received no information regarding (1) whether the BBN transmitters (e.g. physicians) received formal training, (2) the degree to which they applied the acquired competences, and (3) whether they used a certain protocol (e.g. SPIKE) for preparation and documentation. Certainly, this information would be very valid for the interpretation of our data. Despite these limitations, our study constitutes a considerable sample from four different study sites with regard to organization, location and catchment area.

### Clinical implication

Prospective medical personnel should already be sensitized to the BBN-topic within their academic education because the quality of BBN impacts patients’ treatment adherence, coping ability and psychological mindset (Thistlethwaite 2009). As patient-centred, effective and empathic communication is central in BBN contexts (Baile and Aaron 2005; Thistlethwaite 2009; Karger et al. 2017), we advocate that all service providers (e.g. physicians and multidisciplinary teams) acquire these skills and consistently apply this approach.

Applying a guideline such as SPIKES may sensitize, lead and support physicians during BBN (Baile et al. 2000). Furthermore, Singh and Agarwal (Singh and Agarwal 2017) collected several guidelines in a systematic review, which may facilitate physicians’ work in helping patients to receive early, needs-centred support, accessible and professional psycho-oncological care in accordance with national guidelines (Institute of Medicine 2008), and permit shared decision-making or empower processes for patients in their new role. Relationship-building and applying instruments such as communication plans may help assure BBN quality (Rosenzweig 2012).

Monitoring BBN from both perspectives, as part of quality management, may help to identify bottlenecks or weaknesses at the patient and system levels (e.g. psycho-oncological support needs or training needs). Moreover, it may sensitize physicians at the healthcare-provider level (e.g. skills and competences), or decision-makers at the health system level (e.g. allocating infrastructure or resources). Finally, the fostering of structured optimization processes will underscore the topic’s significance.

Studies show that young and experienced oncologists alike acknowledge difficulties in detecting psychological distress in patients (Ford et al. 1994). Furthermore, trauma often may not be apparent during the BBN, instead manifesting hours or even days after the diagnosis (Matthews et al. 2020). Therefore, physicians may invite psycho-oncologists to participate in the BBN or proactively offer psycho-oncological services to newly diagnosed patients. The client-centred approach has been shown to be most effective (Mifflin 1961).

Meanwhile, physicians should also be aware of measures that may prevent their own burnout due to the emotional stress around BBN (Rosenzweig 2012; Hlubocky et al. 2016; Fallowfield and Jenkins 2004). Specific training, simulation and supervision may help BBN transmitters to overcome challenges and maintain a good quality of BBN (Alelwani and Ahmed 2014; Karger et al. 2017; Vitinius et al. 2013). Such capacity building—as well as other related activities—should be based on professional educational principles and informed evidence [(Fallowfield and Jenkins 2004)].

## Conclusion

Because patients’ perceptions of BBN quality are fundamental determinants of their trajectories, adequate BBN delivery skills and consideration of patients’ needs would appear to be essential in offering high-quality, needs-centred care. Although the patients in this study received psycho-oncological support through the isPO programme, it became clear that there is an urgent need for such support beginning from the very moment of diagnosis.

Early acquisition of patient-centred communication skills—and consequently applying existing guidelines (e.g. SPIKES or ABCDE)—is recommended for optimal BBN. Furthermore, we advocate exploring patients’ perspectives on the quality of BBN (e.g. patient outcome interviews) on a regular basis as part of quality management.

## Data Availability

According to the patient informed consent form, the interview data are not available for scientific use by anyone other than the project group members.
